# What We Owe to Experiments on Animals

**Published:** 1904-02-06

**Authors:** Stephen Paget


### HOSPITAL CLINICS AND MEDICAL PROGRESS.

### WHAT WE OWE TO EXPERIMENTS ON ANIMALS.

By STEPHEN PAGET.

### II.—PATHOLOGY, BACTERIOLOGY, AND THERAPEUTICS.

(*Continued from page* 313.)

#### IX.—TYPHOID FEVER (ENTERIC FEVER).

The typhoid-bacillus was discovered in 1880–81, and was obtained in pure culture in 1884. The first protective inoculations were made at Netley Hospital, July–August 1896, on 18 doctors and others who offered themselves. In October, 1897, during an outbreak of typhoid at the Kent County Lunatic Asylum, the protective treatment was offered to the working staff:—

"All the medical staff, and a number of attendants, accepted the offer. Not one of those vaccinated, 84 in number, contracted typhoid fever; while, of those unvaccinated and living under similar conditions, 16 were attacked. This is a significant fact, though it should in fairness be stated that the water was boiled after a certain date, and other precautions were taken, so that the vaccination cannot be said to be altogether responsible for the immunity. Still, the figures are striking." (*Lancet*, March 19th, 1898).

Toward the end of 1898, a large number of inoculations were made among British troops in India, by Wright of Netley. "They were undertaken," he says (*British Medical Journal*, January 20th, 1900), "under conditions which were very far from ideal. In particular, there is reason to suppose that the results obtained may have been unfavourably influenced by a weakening of the vaccine, brought about by repeated re-sterilization." No case was re-inoculated. The results, compiled with the utmost care that was possible, were as follows:—Inoculated 2,835, had 27 cases=0·95 per cent., with 5 deaths=0 2 per cent. Not inoculated, 8,460, had 213 cases =2·5 per cent., with 23 deaths=0·34 per cent.

Later, in 1899, inoculations were made in India, Egypt, and Malta. (See *Army Medical Report*, 1899 and *British Medical Journal*, September 21st, 1901). In India, among 30,353 persons, 4,502 were inoculated, and 25,851 were left without inoculation: among the inoculated there were 44 cases with 9 deaths; among the non-inoculated, 657 cases with 146 deaths. In Egypt, among 4,835 persons, 461 were inoculated; no case of typhoid occurred among them, but the non-inoculated majority had 30 cases with 7 deaths. In Malta 61 were inoculated and 2,456 not inoculated; the inoculated had no cases, the non-inoculated had 17 cases with 5 deaths.

Then came the South African war. What was to be done? Here was a new protective treatment, the latest finding of bacteriology, an imperfect instrument, still under revision: was it to be put back among rabbits and test-tubes, and locked up in the laboratory, or was it to be offered, for what it was worth, to our men? The right thing was done: there is a clear balance of lives saved throughout the war, but nothing more than that. Some account of this half-success is given in "Experiments on Animals"—with other tables showing good results from the Monsall Fever Hospital, Manchester, from Meerut, India, from Egypt and Cyprus (1900), and from the Richmond Asylum, Dublin.

Beside all this, the early and positive diagnosis of the disease has been immeasurably advanced by the discovery of "Widal's reaction." A drop of blood is taken from a case suspected to be typhoid. One-fiftieth part of this drop, or less than that, added to a drop of nutrient fluid containing living typhoid bacilli, causes them to become agglutinated. "The motility of the bacilli is instantaneously or very quickly arrested, and in a few minutes the bacilli begin to aggregate together into clumps, and by the end of the half hour there will be very few isolated bacilli visible." Months, even years, after the fever, this reaction can still be obtained; or even in a baby whose mother had typhoid before it was born. So sure and so general are the practical uses of this test, that these drops of blood are sent hundreds of miles, all over the kingdom, for the judgment of experts. A similar reaction occurs between the blood of certain other fevers and their specific bacilli.

#### X.—MALARIA. YELLOW FEVER.

Bacteriology has proved, past all possibility of doubt, that the mosquito is an intermediate host, between man and man, of the organisms of malaria and of yellow fever. In the mosquito, the germs of malaria, taken from man, work out for themselves that complex and elaborate phase of their amazing lives, whereby they are made a new generation, able to live again in man. In the mosquito, the germs of yellow fever, taken from man, regain that virulence for man which they had just spent on man. These are but two instances, out of many, of the unfathomable mystery of parasitism:—

"It is not enough for the parasites of an individual animal—say a man—to be able to multiply within that individual; but they must also make arrangements, so to speak, for their progeny to enter into and infect other individuals of the same species. They cannot live for ever in one individual. They must spread, in some way or other, to other individuals. Many parasites employ, in various ways, a second species of animal as a go-between. Thus, some tape-worms, and the worms which cause trichinosis, spend a part of their lives in the flesh of swine; they propagate alternately from men to swine, and from swine to men. The blood parasites which cause the deadly tsetse-fly disease among cattle in South Africa are transferred from one ox to another on the proboscis of the ox-biting tsetse-fly. The progeny of the flukes of sheep enter a kind of snail which spread the parasites upon grass. The progeny of the guinea-worm of man enter a water-flea. The progeny of the parasites which cause Texas cattle-fever live in cattle-ticks, and are transferred by the young of these ticks into healthy cattle." (*Ross, Malarial Fever*, 1902).

Experiments on animals, by feeding or by inoculation, were, of course, necessary for the interpretation of all these transmigratory parasites. For instances more recent we have Graham's study of dengue-fever at Beyrout (*New York Medical Record*, February 8th, 1902), Low's and Lefroy's study of filariasis (*British Medical Journal*, June 14th, 1902), and Bruce's admirable study of the African sleeping sickness, and of the evidence of its identity with the tsetse-fly disease (*British Medical Journal*, November, 1903). But, for want of room, all these good works of bacteriology must be left out of account here.

(i.) *Malaria.*

The organism of malaria was discovered by Laveran in 1880. Manson, in 1894, having proved that the mosquito is the intermediate host of filariasis (elephantiasis), brought about a general acceptance of the old idea that the mosquito is a carrier of malaria. Then came MacCallum's observations (1895) at the Johns Hopkins University, and Ross's observations (1895–1898) in India. By three years of laborious microscope-work, with innumerable experiments on mosquitoes and sparrows, Ross discovered and demonstrated the complete cycle of the life of *plasmodium malariæ*, and all its bewildering transformations. From 1898 onwards, Malaria Commissions, from London, Liverpool, and Germany, were poured into Africa or India, one atop of another —it would take an encyclopædia to tell the whole story. In Italy, where this accursed disease kills, or half-kills, legions of poor folk every year, and keeps vast territories of fertile land useless, great things have been done; but there is no room here to tell everything. Let us look at two experiments only :—

(1) In 1900 a wooden hut, made in England, was shipped to Italy and put up near Ostia on a water-logged, jungly bit of country teeming with insect-life and so intensely malarial that all the dwellers there had malarial poisoning, and all farm-labourers who came for the harvest were soon infected. Sambon, Low, Terzi, and two attendants lived in the hut all through the height of the malarial season. They took not one grain of quinine: they were about freely by day, but with an eye for mosquitoes. Netting and curtains were the one and only precaution. And they all remained perfectly well.

(2) In London, New York, Italy, and India, about 1900, men of science let themselves be bitten by infected mosquitoes. The London mosquitoes were sent from Rome, through the British Embassy, to the London School of Tropical Medicine. They had been charged, so to speak, from patients at Rome with "pure benignant tertian." Two men of science (one of them has lately died of yellow fever at Hongkong, where he was studying that disease) exposed their hands and arms to some thirty of the mosquitoes, and in due time had malarial fever, and the tertian type of the parasite was found in their blood.

It is an open question, whether the germs of malaria can live or retain virulence save in man or in the mosquito. Manson is of opinion that they may possibly be able to live from mosquito to mosquito, or in some other intermediate host, or even outside any host. Grassi is of opinion that they have no choice in the matter, and no life outside the one or the other of their alternate hosts. Practically, the campaign against malaria is a campaign against the mosquito. All the world over, from Hongkong to Staten Island, from India to Italy, and on the coast of Africa, war has been declared against this murderous insect; and there are four methods of warfare.

1. To "shut off the sources of infection." That is, to treat all malarial cases, in a given neighbourhood, with quinine, till their blood is so far free from the parasites that the mosquitos biting them do not become infected, and so cannot transmit the disease. This method was followed by Koch, and the German Commission, in New Guinea: a vast number of malarial persons were hunted up and, as it were, sterilized with quinine; and in this way a great reduction of the disease was obtained. The report from Stephansort to the German Government says: "The results of our experiment, which has lasted nearly six months, have been so uniform and unequivocal, that they cannot be regarded as accidental. We may assume that it is entirely owing to the measures we have adopted that malaria here has, in a comparatively short time, almost disappeared." Doubtless, for small communities, this method is of great value; but, as Sir William MacGregor says, it is not applicable to a whole population, *e.g.*, the West Coast of Africa. "In the first place the great mass of natives would not take the medicine, and in the second place the Government could not afford to pay for the 70 tons of quinine a year that would be required to give even a daily grain dose to each of 3,000,000 of people."

2. To keep Europeans apart from natives. This method is based on the fact that practically all native children in Africa contain the parasites of malaria. This fact is abundantly proved by three Commissions. Adult natives may be non-infective, but the vast majority of the children are infective. Therefore European settlements must be kept half a mile from native huts, *i.e.*, outside the mosquito radius. "Each of these huts certainly contains many children with parasites in their blood, and also scores or hundreds of mosquitoes to carry the infection." Unhappily, in Africa, European barracks, factories, houses, and railway buildings, are put anywhere and anyhow, with native huts, full of children and mosquitoes, huddled right up against them. Commerce, business, custom, and goodwill among men, are all against any violent or universal boycotting of natives, and absolute segregation is hardly possible. Still, a great deal can be done; and Heaven knows there is plenty of evidence that it ought to be done. (See *Report of the Nigeria Expedition, Liverpool School*, 1900.)

3. To keep off the mosquito. Wire-netting to the windows and the chimneys, mosquito-nets at night, and a knowledge of the haunts and habits of the insect, are a great safeguard: witness, Sambon's and Low's experiment near Ostia, and Grassi's experiment, on a much wider scale, on the Battipaglia-Reggio railway. But the African native cannot be got to understand these things: and the Anglo-African, unlike the Anglo-Indian, is neglectful of his health, and is often ill-fed, ill-housed, and ill-exercised: a very dismal life, intolerant of the additional worry of nets and netting. As Sir William MacGregor says, "It is not likely that in a place like Lagos as good results can be obtained from the use of mosquito-proof netting as in Italy. In a low-lying, hot, and moist locality like Lagos, it comes to be a choice of evils, to sit inside the netting stewed and suffocated, or to be worried and poisoned by mosquitoes outside. Few officers or others are so occupied that they could spend the day in a mosquito-proof room. Operations here are being limited to supplying one mosquito-proof room to the quarters of each officer. The European wards of the hospital are similarly protected."

4. To keep down the mosquito. Its breeding-places are stagnant or semi-stagnant puddles, swamps, or small ponds, cisterns, and the rain-water that is caught by barrels, empty tins, old bottles, broken crockery, and all such dust-heap rubbish. The drainage or stocking of stagnant ponds, the filling up of puddle-holes, the covering of barrels and cisterns, the carting away of all broken odds and ends that can hold water—these jobs, which can be done by gangs of unskilled labourers and scavengers, abolish the breeding-places, destroy the eggs and larvæ of the mosquito, and break the cycle of its life. See, for a good record of all this good work, two little essays by Ross, published in 1902. (i) *Malarial Fever, its Cause, Prevention, and Treatment.* (ii) *Mosquito Brigades and how to Organise them.*

Thus, out of experiments on mosquitoes and small birds, and a few voluntary self-inoculations among men, has come the present world-crusade against malaria. All the four methods of this warfare, and all better methods that the future may have in store, go straight back to the discovery that malaria is transmitted, after an elaborate series of changes in the body of a mosquito, from man to man.

(ii.) *Yellow Fever.*

The earliest experiments on yellow fever were made about 1815 by Chervin, Potter, Firth, Catteral, and Parker on themselves. These experiments were of the most appalling severity, and of no value whatever. In 1880 the Board of Health and the Navy Department of the United States published reports tending to show that yellow fever was a "germ-disease," and that it could increase in virulence, apart from man, in itself, and of its own accord. Finlay, on these reports, based his theory that the disease was transmissible, through insects, with sustained or increased virulence, from man to man:—

"I conceived the idea that the yellow-fever germ must be conveyed from the patient to the non-immunes by inoculation, a process which could be performed in nature only through the agency of some stinging insect whose biological conditions must be identical with those which were known to favour the transmissibility of the disease."

In the words of Claude Bernard, *This was a theory, that is to say, an idea leading me to make experiments.* Therefore, in 1881, Finlay, and six others, let themselves be bitten by infected mosquitoes, and obtained mild attacks of the fever, and some subsequent immunity. Between 1881 and 1900 he inoculated 104 persons by this method, taking care that the insects should come from mild cases of the disease, and that no time should be given for any increase of its virulence while it was in the mosquito. Most of these inoculations failed to produce even the slightest touch of fever; but they did confer more or less immunity. Still, men will not submit themselves gladly to a mosquito charged with yellow fever, even though it be warranted mild. In 1896 Sanarelli discovered the *bacillus icteroides*; in 1897 he succeeded in immunising animals against it; in 1898 he got very good results, preventive and curative, with a serum treatment. It is to be noted against him that he made five experiments on human beings, two of whom were condemned criminals. During 1901 and 1902 valuable Reports were issued by two American Commissions, and by a Commission of the Liverpool School. (Two Commissioners, Dr. Lazear and Dr. Walter Myers, became infected with the disease and died of it.) These Commissions did for yellow fever what Ross had just done for malaria. As Ross had identified one species of *Anopheles* in malaria, so they identified one species of *Culex* in yellow fever. The haunts and habits of *Culex*, and its breeding places, which are different in some ways from those of *Anopheles*, have been studied with infinite care; and the measures which tend to keep out and keep down the one are also efficacious against the other. See here the 1902 report by Surgeon-Major Gorgas, chief sanitary officer, Havana; it is quoted in abstract in *The Practitioner, May* 1902:—

"Commencing in February 1901, orders were issued that every suspected case of yellow fever should be screened with wire gauze at the public expense, so as to render the room or rooms mosquito-proof. All mosquitoes in the infected house and in contagious houses were destroyed. After the middle of February, 100 men were employed in carrying out the destruction of the larvæ in their breeding places, putting oil in the cesspools of all houses, clearing the streams, draining pools, and oiling the larger bodies of water. Up to June quarantine was enforced, together with disinfection of the house and fomites. After that, however, rigid quarantine of the patient was stopped, and disinfection of fabrics and clothing ceased . . . . *By the end of September the last focus of the disease had been got rid of, and since then up to the beginning of January there has not been a single case. For the first time in* 150 *years Havana has been free from yellow fever.*"

---

"Thus, in a few years," says "Experiments on Animals," from experiments on mosquitos, sparrows, and men, has come the present plan of campaign against malaria, yellow fever, and filariasis; that is, against *Anopheles* and *Culex.* There has been nothing like it since Pasteur died. Far and wide, from Staten Island to Cuba, from Hongkong to Lagos, the work of keeping down the larvæ of *Anopheles* and *Culex* is going on. *Henceforth we have to reckon not with a nameless something, but with a definite parasite, whose conditions of life are known. Before all things, we must shut off the sources of the infection.* For centuries, men had believed in exhalations and miasmata lying all over the land; and, behold, the agents of malaria are in the puddles round a man's house, and the agents of yellow fever are in the water-butt and the broken bottles and old sardine tins. Science has given the word, and now there are *Anopheles* brigades and *Culex* brigades set going; labourers with brooms and rubbish carts, sweeping out the stagnant pools, draining the surface soil, and carrying off the odd receptacles that serve to hold mosquito eggs and larvæ. The job, like all sanitary jobs, must be steady, year in and year out; it must be limited to infected places, a whole continent cannot be treated. But there the work is, and will grow; and saves, by unskilled labour, and at a trivial expense, those "non-acclimatised" lives that have hitherto been thrown away."

Summary of Experiments in Pathology, etc.

As in physiology, so in pathology, many examples have been omitted here for want of space. Nothing has been said of the treatment of myxœdema and sporadic cretinism with thyroid substance, or of the work of Magendie and Bernard on the selective action of drugs, or of other experiments in pharmacology, or of Calmette's and Fraser's work on snake-venom and antivenene, or of Hunter's recent work on pernicious anæmia. Nothing has been said of many excellent methods that we owe indirectly, but not directly, to experiments on animals: for instance, the uses of electricity in the diagnosis and treatment of injuries and diseases of the nerves and nerve centres. Let us leave all these present gains, and look into the near future. Take diphtheria, or take tetanus. All further knowledge of them, all better treatment of them, will come out of our present knowledge and our present treatment, which have come out of experiments on animals. Take cancer. Is it possible that any research into this vast group of diseases should lead to practical results without the help of these experiments? Take rheumatic fever, or take ulcerative endocarditis. Can anybody doubt that the best hope of a better treatment of these diseases is in bacteriology? Everywhere, in every civilised country, the experts are hard at work in the unity of science. *Securus judicat orbis terrarum*: the whole world is of one mind here, and one faith.

(*To be continued.*)

